# Contralateral lymph node metastasis in a woman with new primary breast cancer: Systemic desease or locoregional diffusion?

**DOI:** 10.1016/j.ijscr.2018.11.001

**Published:** 2018-11-14

**Authors:** Angela Strazzanti, Santi Gangi, Claudio Trovato, Nicola Pacini, Francesco Basile

**Affiliations:** a Universita degli Studi di Catania, Scuola di Facolta di Medicina, Surgery Catania, 95124, Sicily, Italy; b Universita degli Studi di Catania, Scuola di Facolta di Medicina, General Surgery Catania, 95124, Sicily, Italy; c Azienda Sanitaria Provinciale di Reggio Calabria, Biochemistry Reggio Calabria, 89125, Calabria, Italy

**Keywords:** MRI, magnetic resonance imaging, IBR, ipsilateral breast reccurence, CLNs, contralateral lymph-nodes, Contralateral axillary lymph node metastasis (CAM), Skin invasion, Multifocal cancer, Locoregional extension of the disease

## Abstract

**Introduction:**

Contralateral axillary lymph node metastases (CAMs) in patients with breast cancer are rare (Daoud et al., 1998); however, CAMs may be already detected at the time of primary breast cancer diagnosis (synchronous CAM) or after a previous treatment of breast cancer as a recurrence if not as an ipsilateral breast recurrence (IBR) (metasynchronous CAM) (Zhou and Richir, 2013).

The involvement of the contralateral axilla could be caused by a systemic disease (stage IV), a regional metastasis from a new occult primary tumor (T0N1, stage II) or a breast cancer recurrence It may also arise from a locally advanced disease in a patient who has suffered from a primary breast cancer.

**Presentation of Case:**

This report focuses on the case of a 50-year-old woman who has developed a new primary breast cancer, breast skin invasion and CAMs.

**Discussion:**

We intend to show that an altered lymphatic drainage may result from CAMs; in fact, patients who have previously undergone axillary lymph node dissection (ALND) are more likely to develop contralateral lymph drainage (Maaskant-Braat et al., 2013).

**Conclusion:**

Along with that, we want to support the theory that CAMs should be treated with curative intent rather than as a stage IV disease, as we believe that CAMs are due to a locoregional extension of the disease.

## Highlights

• Contralateral axillary lymph node metastases (CAMs) in patients with breast cancer are rare.
• CAMs should be considered as a regional manifestation, rather than systemic disease.
• The survival of CAM cannot be compared to a distant disease.
• Most patients receive locoregional and systemic treatment according to a curative approach.

## 1 Introduction

Contralateral axillary lymph node metastasis (CAM) is rather unusual with literature citing an incidence ranging from 1.9 to 6 per cent [[1], [2], [3], [4]].

The rarity of contralateral axillary lymph-node metastasis (CAM) has been a controversial topic. Moreover, the uncertain laterality of the cancer responsible for this metastasis complicates the overall disease staging and is a management dilemma for clinicians.

There remains controversy over whether CAMs should be considered distant metastases or locoregional spread. It is also uncertain why or how CAM occurs. It mainly arises from a relapse after the treatment of the primary tumor with aggressive histopathological features and in the absence of metastatic disease elsewhere.

In addition to the aggressive histopathological features, an altered lymphatic spread from the tumor to the contralateral axilla contributes to the development of CAM. The development of alternative routes of lymphatic drainage might be prompted by damage to the usual draining lymphatics.

Irradiation or previous axillary surgery, for example, can cause it [[2], [3], [4], [5]].

Haagensen hypothesized that tumor cells could spread to the contralateral axillary nodes by permeating through the deep lymphatic plexus of the chestwall [6].

After curative treatment for breast cancer, a small proportion of patients experience a contralateral lymph node recurrence or secondary ipsilateral breast cancer during follow-up. When CAMs are present at initial diagnosis, contralateral lymph-nodes (CLNs) are traditionally considered to be a result of systemic dissemination. No classification for CAM is found in the most recent version of the AJCC Cancer Staging Manual, where it used to be classified as a distant disease.

However, lymphoscintigraphy studies in patients who have previously undergone surgery of the breast or axilla show altered lymphatic drainage to contralateral lymph-nodes, such as the contralateral axilla, internal mammary chain or periclavicular sitesm [[7], [8], [9], [10], [11], [12], [13], [14]].

Hypothetically, these aberrant drainage patterns might indicate that CAMs, after previous treatment for breast cancer, should be considered as a regional manifestation, rather than systemic disease from the breast via lymphatics rather than hematogenous spread.

### 1.1 Clinical case

Herein we report the case of a Caucasian 50-year-old woman who had been operated in 2013 for a 18 mm breast invasive ductal cancer which was poorly differentiated.

The initial breast cancer was located in the external upper right quadrant; it was negative for hormone receptors, HER- 2/neu overexpressing, ki67 30%, and associated with lymphovascular invasion. First the patient underwent a right side quadrantectomy and lymphoadenectomy for a lymph node metastasis (3/10). Then she underwent a total body CT scan without contrast, because she was allergic to the contrast. Finally, a bone scintigraphy was also carried out. In the end, no body metastases were found. According to Saint Gallen criteria she was a pT1cN1aM0.

From a genetic test, the patient did not present a germ line mutation for BRCA 1/2.

After one month she started receiving adjuvant chemotherapy. The patient underwent a 4-cycle treatment with AC then taxolo treatment x 12 weeks and herceptin x 54 weeks. In addition to that she underwent local radiation therapy.

The follow up surveillance showed good general conditions until December 2017.

In January 2018 the patient reported about the appearance of local cutaneous nodes in the right breast, that were merely diagnosed as skin lesions. The increase of lesions despite the topical therapy made the patient decide to ask us for a second opinion. At the clinical assessment we assumed the presence of multiple cutaneous metastases in the breast.

The patient underwent 18F-fluorodeoxyglucose positron emission tomography/computed tomography (FDG PET/CT) for staging work-up. Owing to her allergy to the contrast, it was impossible to carry out a total body CT or breast MRI. The FDG PET/CT showed increased FDG uptake in the right breast, in the omolateral internal mammary chain, in the left axillary lymph nodes and left subclavicular lymph nodes.

Consequently, it was useful to perform a the FDG PET/CT and lymphoscintigraphy to detect unpredictable contralateral axillary lymph node metastases from a second primary breast cancer. Later in May, she underwent right rescue mastectomy and left mastectomy (the latter at her request and in order to discover an eventual CUP-syndrome) together with a left lymphadenectomy. But she refused recostruction with prosthesis.

The final pathological diagnosis revealed a right side poorly differentiated multiphocal breast invasive ductal cancer, the biggest lesion being 1 cm, with extensive dermal angiolymphatic diffusion. The tumor was ER/PR negative, Her-2 positive as before and ki67 was 10%.

It only revealed a cystic fibrosis in the left breast and no evidence of tumor.

As regards the left axilla the final pathological diagnosis showed a bigger and palpable lymph node and 2 additional subclavicular lymph nodes which were positive for metastases and 5 additional axillary lymph nodes which resulted negative. Prognostic factors in axillary lymph nodes were ER/PR negative, Her-2 positive and ki67 was 60%.

## 2 Discussion

There has been conflict in literature whether CAM should be considered as a distant disease and thus treated as a stage IV breast cancer rather than a locoregional extension of contralateral breast cancer. Breast cancer metastasis in ipsilateral supraclavicular lymphnode is assigned a N3 status in the TNM system and thus classified as stage III disease in the American Joint Commissionon Cancer Staging Manual.

Breast cancer metastasis in the contralateral axillary lymph node (CAM) with no metastasis in any other distant organ is currently assigned M1 status (stage IV) instead of N3 (stage III) [15]. When a cancer is found in the contralateral axilla, there are three main potential sources [18]: a)contralateral spread from the original breast tumor; b) metastasis from an occult primary tumor in the ipsilateral breast or c) metastasis from an extramammary site. The sources include the adenocarcinoma of the uterus, of the gastrointestinal tract, ovary, thyroid, kidney; lymphoma or melanoma; squamous cell cancer from the lung or skin; a primary tumor of the sweat gland; or a neurogenic tumor [16,17].

Management complicates when CAM occurs as a result of the first event of relapse after treatment of the primary tumor, especially in the absence of metastatic disease elsewhere.

Lymphoscintigraphy studies for IBR performed on the patient before breast/axillary surgery have shown new drainage patterns to contralateral axilla.

The real incidence of CAM is difficult to estimate. On one hand, the rates may be overestimated due to the lack of MRI for the diagnosis of occult contralateral tumors. On the other hand, the incidence may be underestimated because patients may happen to ignore physical exam findings or fail to have adequate follow-up. In our case we did not perform MRI or CT because our patient was allergic to the contrast. We carried out a FDG PET/CT and lymphoscintigraphy to detect unpredictable contralateral axillary lymphnode metastases from a second primary breast cancer.

Previous studies indicate that patients with contralateral axillary lymph node metastases (CAM) have a better outcome compared to patients having a stage IV disease [15].

This study has been undertaken to evaluate the clinicopathological characteristics of the tumor and the outcome of patients who had developed CAM without any other distant metastatic disease at the time of CAM diagnosis.

## 3 Conclusion

Given the angiolymphatic diffusion and the multiple metastases in the medial quadrant of the right breast and the absence of distant metastases, we believe that it is possible to hypothesize a presumable “inversion of the lymphatic flow” consequent to the lymphadenectomy, which results into a "crossover" lymphatic drainage through the lymphatics of the sternal region or the deep internal mammary chain, already involved. As three weeks after mastectomy, the appearance of further skin recurrences occurred again, it follows that we can consider CAM as a locoregional disease, and therefore a surgical treatment with healing intent is justified. We think that surgical treatment is the right option if we consider CAM as a loco-regional deasease or secondary site of a Cup-syndrome.

Therefore, we recommend designating CAM as N3 and not as M1 in the future AJCC staging manual.

The survival of CAM cannot be compared to a distant disease; consequently, most patients receive locoregional and systemic treatment according to a curative approach.

To conclude there is evidence that CAM should be regarded as a regional event [19].

Our work has been reported in line with the SCARE criteria [20]

## Conflict of interest

There is no conflict of interest with any of the authors involved in this paper.

## Funding source

None.

## Ethical approval

No ethics approval was needed.

## Consent

Written informed consent was obtained from the patient for publication of this case report and accompanying images. A copy of the written consent is available for review by the Editor-in-Chief of this journal on request.

## Author contribution

Trovato Agata: Literature review and writing the article.

Nicola Pacini MD: Reviewing and editing the article.

## Registration of research studies

None.

## Guarantor

Prof. Francesco Basile

## Provenance and peer review

Not commissioned, externally peer reviewed.
