# Supplementary material for: Contralateral lymph node metastasis in a woman with new primary breast cancer: Systemic desease or locoregional diffusion?
Source: Int J Surg Case Rep. 2018 Nov 14;53:400–2. doi: 10.1016/j.ijscr.2018.11.001 (PMC6259044; doi:10.1016/j.ijscr.2018.11.001)
Supplement: Supplementary file 1 [file mmc1.pdf]

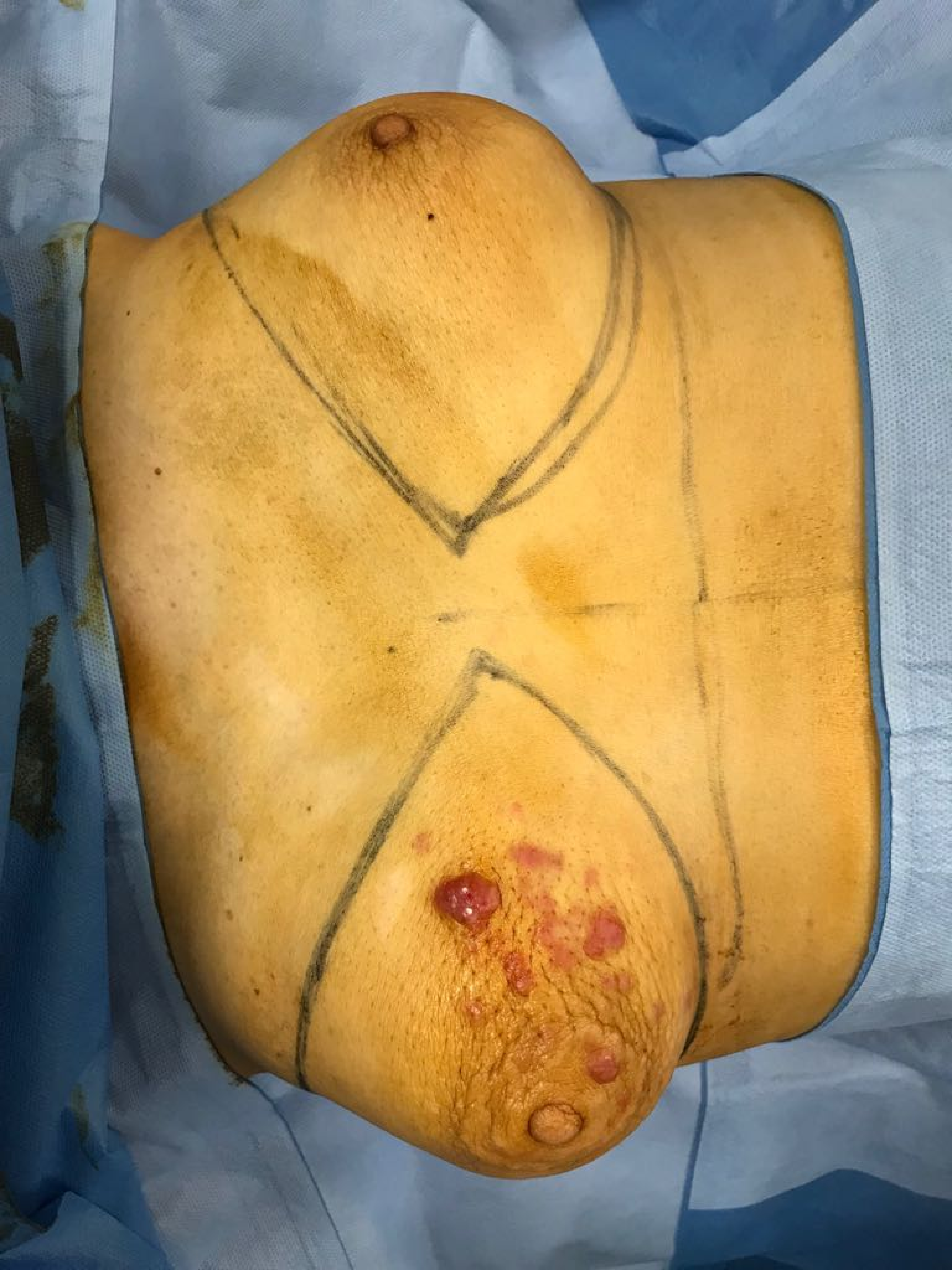

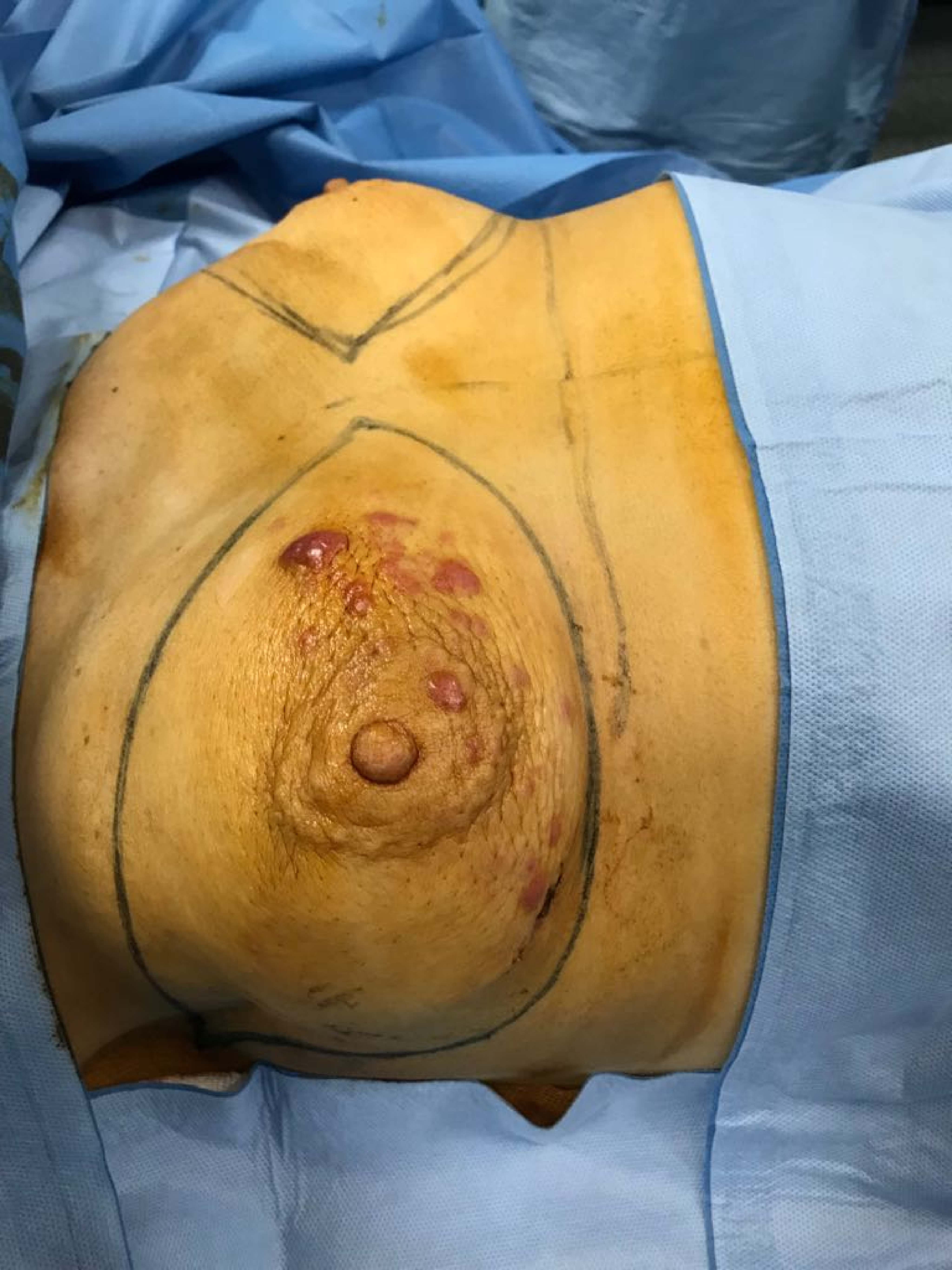

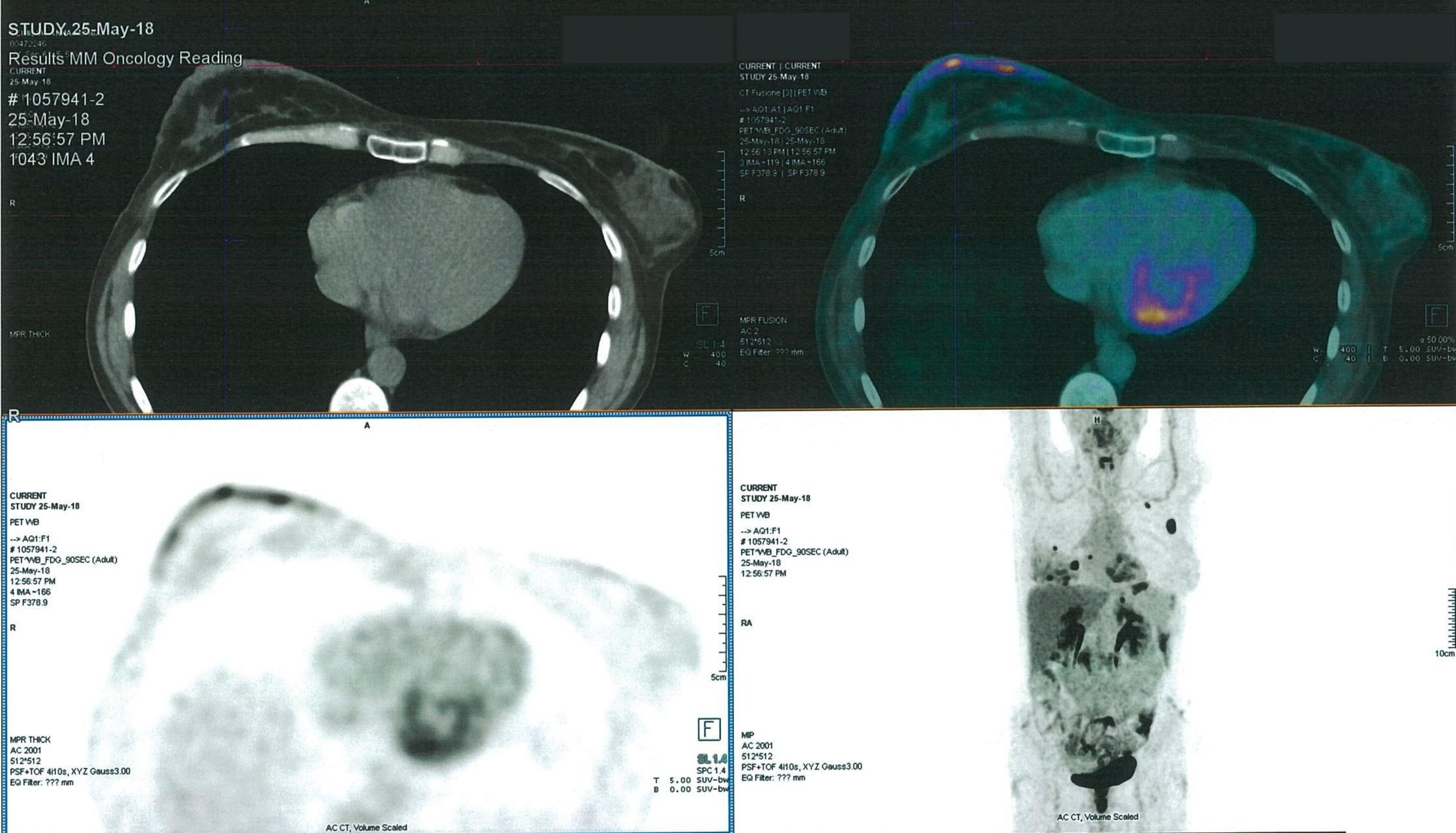
STUDY 25-May-18
Results MM Oncology Reading
CURRENT
25-May-18
1057941-2
25-May-18
12:56:57 PM
1043 IMA 4
R
MPR THICK
5cm
F
SL 1.4
W 400
C 40
CURRENT | CURRENT
STUDY 25-May-18
CT Fusione [3] | PET WB
--> AQ1:A1 | AQ1:F1
1057941-2
PET^WB_FDG_90SEC (Adult)
25-May-18 | 25-May-18
12:56:13 PM | 12:56:57 PM
3 IMA ~119 | 4 IMA ~166
SP F378.9 | SP F378.9
MPR FUSION
AC 2
512*512
EQ Filter: ??? mm
α 50.00%
W 400 T 5.00 SUV-bw
C 40 B 0.00 SUV-bw
A
CURRENT
STUDY 25-May-18
PET WB
--> AQ1:F1
1057941-2
PET^WB_FDG_90SEC (Adult)
25-May-18
12:56:57 PM
4 IMA ~166
SP F378.9
MPR THICK
AC 2001
512*512
PSF+TOF 4i10s, XYZ Gauss3.00
EQ Filter: ??? mm
SL 1.4
SPC 1.4
T 5.00 SUV-bw
B 0.00 SUV-bw
AC CT, Volume Scaled
H
CURRENT
STUDY 25-May-18
PET WB
--> AQ1:F1
1057941-2
PET^WB_FDG_90SEC (Adult)
25-May-18
12:56:57 PM
RA
MIP
AC 2001
512*512
PSF+TOF 4i10s, XYZ Gauss3.00
EQ Filter: ??? mm
10cm
AC CT, Volume Scaled

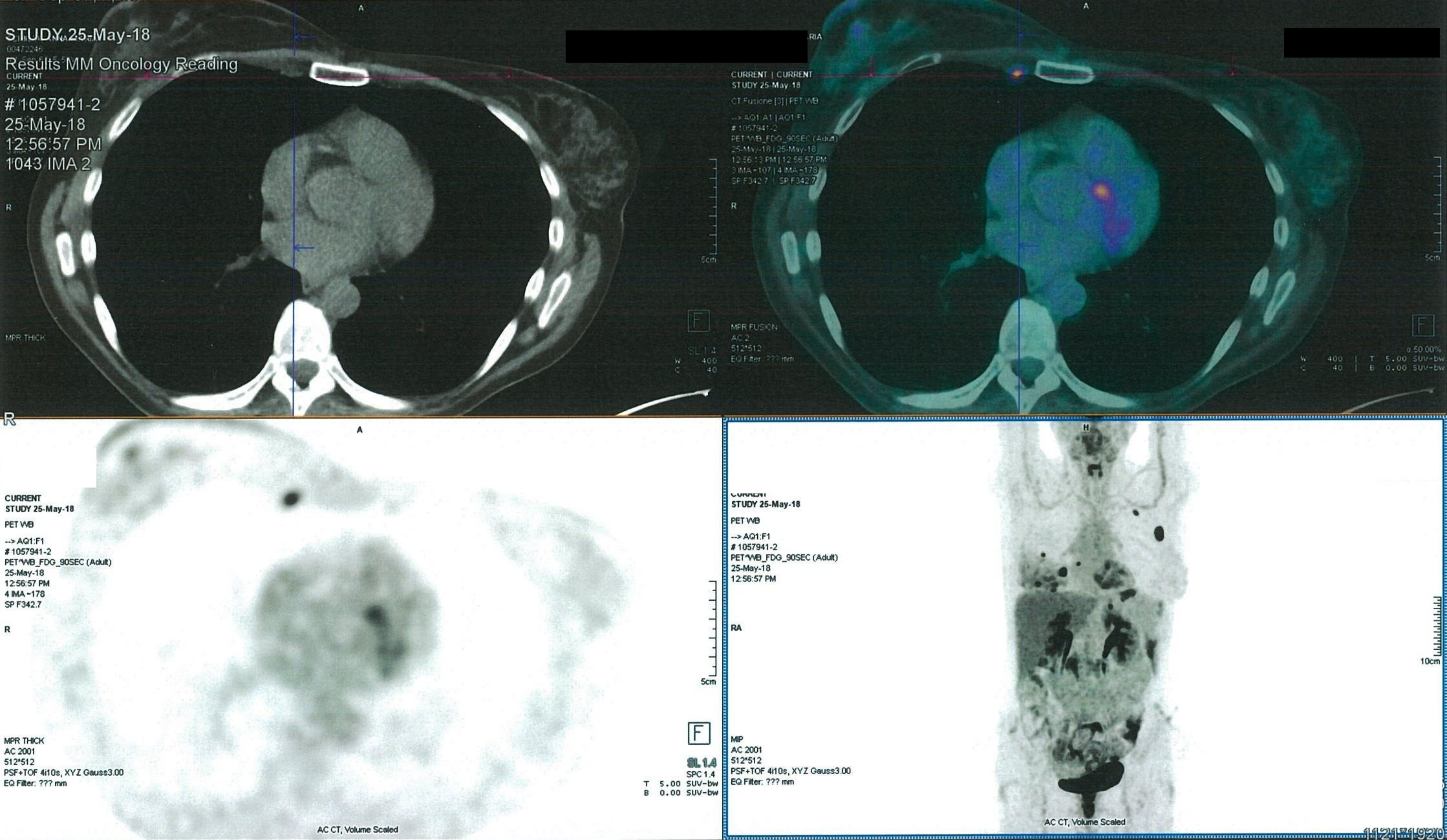
STUDY 25-May-18
Results MM Oncology Reading
CURRENT
25-May-18
1057941-2
25-May-18
12:56:57 PM
1043 IMA 2
A
R
[redacted]
5cm
MPR THICK
SL 1.4
W 400
C 40
CURRENT | CURRENT
STUDY 25-May-18
CT Fusione [3] | PET WB
--> AQ1:A1 | AQ1:F1
1057941-2
PET^WB_FDG_90SEC (Adult)
25-May-18 | 25-May-18
12:56:13 PM | 12:56:57 PM
3 IMA -107 | 4 IMA -178
SP F342.7 | SP F342.7
[redacted]
MPR FUSION
AC 2
512*512
EQ Filter: ??? mm
α 50.00%
W 400 | T 5.00 SUV-bw
C 40 | B 0.00 SUV-bw
CURRENT
STUDY 25-May-18
PET WB
--> AQ1:F1
1057941-2
PET^WB_FDG_90SEC (Adult)
25-May-18
12:56:57 PM
4 IMA -178
SP F342.7
MPR THICK
AC 2001
512*512
PSF+TOF 4i10s, XYZ Gauss3.00
EQ Filter: ??? mm
SL 1.4
SPC 1.4
T 5.00 SUV-bw
B 0.00 SUV-bw
AC CT, Volume Scaled
H
STUDY 25-May-18
PET WB
--> AQ1:F1
1057941-2
PET^WB_FDG_90SEC (Adult)
25-May-18
12:56:57 PM
RA
10cm
MIP
AC 2001
512*512
PSF+TOF 4i10s, XYZ Gauss3.00
EQ Filter: ??? mm
AC CT, Volume Scaled
1121*1920

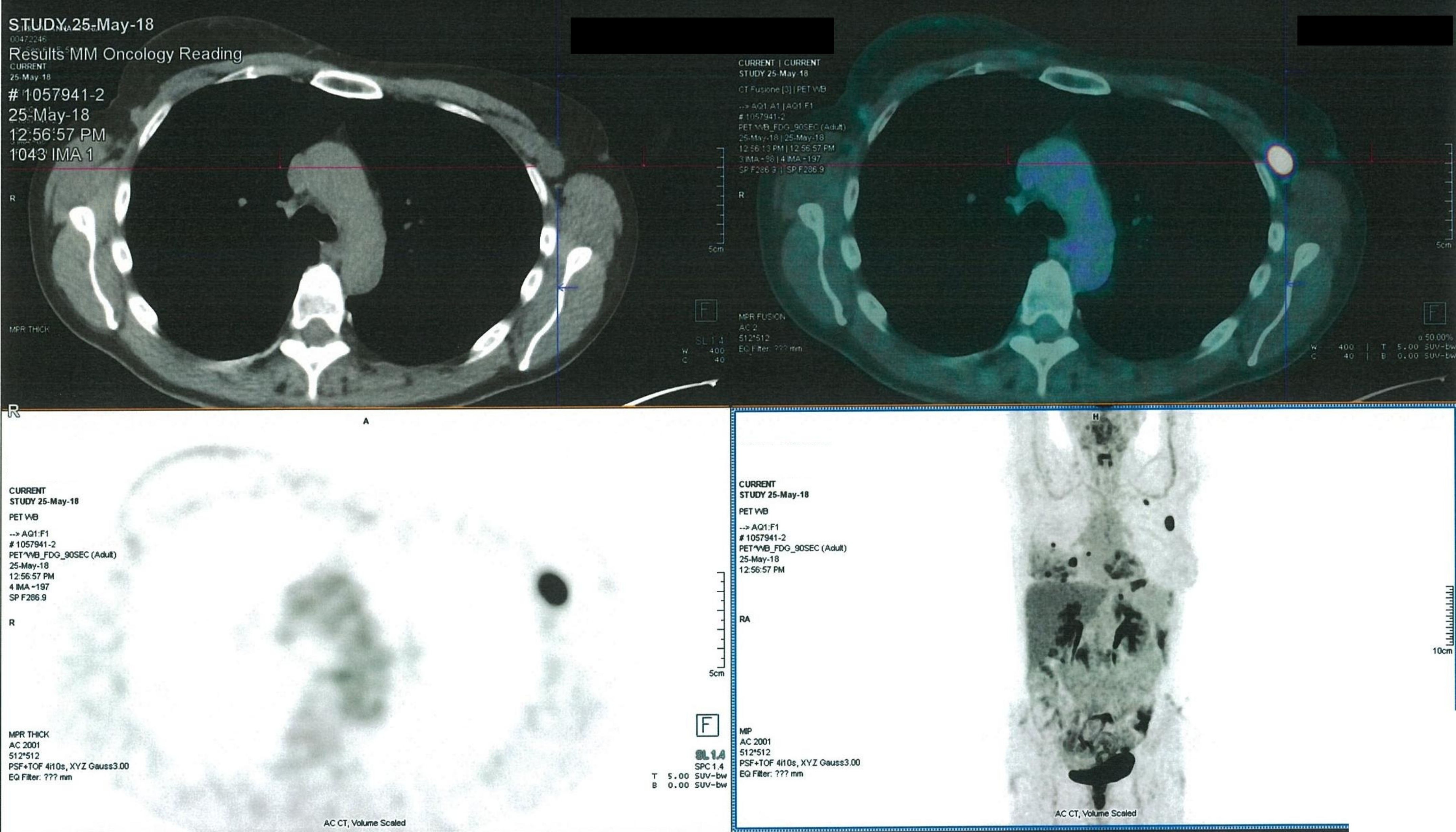

STUDY 25-May-18
Results MM Oncology Reading
CURRENT
25-May-18
1057941-2
25-May-18
12:56:57 PM
1043 IMA 1
R
5cm
F
MPR THICK
SL 1.4
W 400
C 40
CURRENT | CURRENT
STUDY 25-May-18
--> AQ1:A1 | AQ1:F1
1057941-2
PET WB_FDG_90SEC (Adult)
25-May-18 | 25-May-18
12:56:13 PM | 12:56:57 PM
SP F286.9 | SP F286.9
MPR FUSION
512*512
EQ Filter: ??? mm
W 400 | T 5.00 SUV-bw
C 40 | B 0.00 SUV-bw
R
A
CURRENT
STUDY 25-May-18
PET WB
--> AQ1:F1
1057941-2
PET WB_FDG_90SEC (Adult)
25-May-18
12:56:57 PM
4 IMA -197
SP F286.9
R
5cm
F
MPR THICK
AC 2001
512*512
PSF+TOF 4i10s, XYZ Gauss3.00
EQ Filter: ??? mm
SL 1.4
SPC 1.4
T 5.00 SUV-bw
B 0.00 SUV-bw
AC CT, Volume Scaled
H
CURRENT
STUDY 25-May-18
PET WB
--> AQ1:F1
1057941-2
PET WB_FDG_90SEC (Adult)
25-May-18
12:56:57 PM
RA
10cm
MIP
AC 2001
512*512
PSF+TOF 4i10s, XYZ Gauss3.00
EQ Filter: ??? mm
AC CT, Volume Scaled
